# Chemical Characterization and Phenolic Profiling of Commercial Olive Leaf Extracts Using LC-Q-TOF-MS and HPLC-DAD

**DOI:** 10.3390/foods15203666

**Published:** 2026-10-15

**Authors:** Zeliha Ustun-Argon

**Affiliations:** 1 Department of Medical and Aromatic Plants, Meram Vocational School, Necmettin Erbakan University, Konya 42090, Türkiye; zuargon@gmail.com; 2 Medicinal and Cosmetic Plants Application and Research Center (TİBAM), Necmettin Erbakan University, Konya 42140, Türkiye

**Keywords:** *Olea europaea*, olive leaf extract, oleuropein, LC-Q-TOF-MS, HPLC-DAD, phenolic compounds, commercial supplements, phytochemical profiling

## Abstract

This study comparatively evaluated the chemical fingerprints and oleuropein contents of commercially available olive leaf extracts on the Turkish dietary supplement market. Six independent commercial products, comprising three liquid and three powder/capsule formulations, were characterized by LC-Q-TOF-MS, while oleuropein was quantified by targeted HPLC-DAD analysis. Among the LC-Q-TOF-MS annotations, 288 compounds were operationally classified as phenolic or phenolic-related compounds. Of these, 114 were annotated only in liquid products, 113 only in powder/capsule products, and 61 in both groups. Powder/capsule products showed greater phenolic-class richness, particularly in flavonoid annotations. HPLC-DAD confirmed oleuropein in all six products, although concentrations varied markedly among samples. However, because the products were independent commercial preparations, these differences cannot be attributed solely to physical form. Overall, combining LC-Q-TOF-MS profiling with targeted HPLC-DAD quantification provided complementary information on phytochemical heterogeneity and product-to-product variability in commercial olive leaf extracts.

## 1. Introduction

One of the major agricultural crops from the Mediterranean region is the olive (*Olea europaea* L.), both economically and culturally, and there is a significant amount of foliage generated through the process of growing and producing olive oil. Though for many years olive leaves were considered a low-value agricultural waste, more recently, olive leaves became an interesting subject for investigations regarding their ability to act as functional foods and provide food supplements or bioactive compounds due to the high content of phenolic compounds [1,2]. Olive leaves contain different types of compounds, including secoiridoids, phenolic alcohols, phenolic acids, flavonoids, and phenylpropanoid derivatives. Oleuropein is one of the main phenolic compounds contained in the leaves; however, other phenolic compounds like hydroxytyrosol and its derivatives, tyrosol, verbascoside, ligstroside, elenolic acid derivatives, luteolin and apigenin glycosides, rutin, and quercetin are often found in the phenolic content of olive leaves [2,3,4]. Cynaroside (luteolin-7-glucoside), quinic acid, oleuropein, and verbascoside were also identified as the main compounds in Nizip oil olives [3].

Nevertheless, the phenolic content of olive leaves varies and is dependent on a number of factors such as the genotype, geographical location, and environmental conditions [5,6]. Investigations on various cultivars grown in different environments have shown that oleuropein, verbascoside, and flavonoids play an important role in the chemical distinction of the leaves [5,7]. Palm et al. showed that stress conditions may affect the profiles of various polyphenols, including quercetin, rutin, oleuropein, and luteolin, in different genotypes of *O. europaea* [6]. In addition, the extraction technique, the solvent applied, the temperature, and the drying procedure affect the phenolic compounds as well [8,9,10]. This means that the chemical profile of the olive leaf extract should be evaluated not only in regard to the plant material but also the processing of the raw material.

The above variation is particularly significant in the context of commercially available olive leaf products. According to Medina et al., the amount of bioactive molecules in commercial olive leaf extracts showed substantial variations between the products, and oleuropein is the dominant phenolic compound in the tested products [11]. The commercial extracts may be available in different physical forms such as liquids and powders, and the process of extraction, dehydration, and storage may affect the characteristics of the final product. Ahmad-Qasem et al. found that the dehydration of the olive leaf extracts causes an about 10 percent decrease in total phenolic content and antioxidant activity; however, in the context of controlled storage, the physical form did not significantly affect phenolic content on its own [12]. Likewise, it has been found that processes like the drying and milling of olive leaf play an important role in the preservation of the bioactive components and extraction process [9,10]. Thus, the chemical distinctions made by different types of liquid and powders must not be considered due to their physical form but rather the different phytochemical profile, which also includes their composition and manufacture.

The identification of complex natural plant extracts is facilitated through high-resolution mass spectrometry, which provides a highly effective analytical technique for the screening of a vast number of metabolites in a single experiment. Specifically, the combination of HPLC or LC with electrospray ionization quadrupole time-of-flight mass spectrometry (ESI-Q-TOF-MS) has led to a comprehensive profiling of secoiridoids, flavonoids, phenols, and phenolic acids in olive leaves using accurate mass, molecular formula, isotopic pattern, and tandem mass spectrometry [13,14,15]. Quirantes-Piné et al. observed the presence of 48 components in olive leaves by HPLC-ESI-QTOF-MS and identified 31 of them as phenolic compounds [13]. Later works confirmed that oleuropein, oleoside/secologanoside isomers, elenolic acid glucosides, ligstroside, and different flavonoid glycosides are important constituents of the specific high-resolution mass spectrum of olive leaves [4]. Nevertheless, the fact that identifications of compounds performed via the matching to databases and spectral libraries in large-scale LC-Q-TOF-MS analysis are tentative should not be forgotten. So, in addition to the chemical profiling, quantification of selected phenolic compounds can contribute to a complete picture of their analysis.

The objective of this research is the comparative characterization of the chemical profiles of six different commercially available preparations of olive leaf extracts—in three liquid and three powder/capsule forms—from the Turkish domestic market. Within this context, comprehensive chemical profiles of the preparations were characterized via LC-Q-TOF-MS analyses in positive and negative ionization modes; comparisons among the commercial preparations were made, focusing on phenolic compound classes and phenolic compounds found in the literature to be present in the *O. europaea* leaf. Moreover, the goal of this study is to discover phenolic compounds that have either similar distribution patterns or ones specific to the preparation group in the liquid and powder/capsule preparations, as well as to quantify oleuropein via HPLC-DAD. Therefore, the aim was to unravel the phytochemical diversity present in commercially available olive leaf extracts by means of an integrated strategy that involves mass spectrometry-based profiling and target chromatography analysis.

## 2. Materials and Methods

### 2.1. Commercial Olive Leaf Extract Samples

Six commercial olive leaf extracts that are commercially available in the Turkish dietary supplements market were used in this study. The commercial preparations were divided into two categories depending on the physical form of the samples. Three liquid preparations (S1–S3) and three hard capsules with powdered contents (S4–S6) were utilized in this study. Every sample represents an individual commercial preparation; thus, the liquid and powdered/capsule samples do not represent paired preparations of the same crude material. Because of the lack of standardization regarding the production conditions such as the manufacturer, type of olives used, harvest period, extraction technique, extraction solvent, dry matter content, carrier substances, etc., possible differences between the liquid and powder/capsule preparations could not be considered as the effect of the physical form independently.

### 2.2. LC-Q-TOF-MS Based Chemical Profiling

Analysis using high-resolution accurate-mass LC-Q-TOF-MS was performed to determine the comprehensive chemical compositions of commercial olive leaf extracts. LC-Q-TOF-MS analyses were performed with the aim of qualitative chemical profiling and not quantitative measurements of individual compounds. As such, the LC-Q-TOF-MS signals or peaks were not assumed to be compound concentrations.

Pure water was obtained from a Milli-Q system (Millipore, Bedford, MA, USA). LC-MS-grade acetonitrile and methanol and the oleuropein reference standard were purchased from Sigma-Aldrich (St. Louis, MO, USA), while formic acid, phosphoric acid, and 0.45 µm PTFE membrane filters were obtained from Merck (Darmstadt, Germany).

#### 2.2.1. Chromatographic Conditions

Chromatographic separation was performed on an Agilent 1260 Infinity series HPLC instrument (Agilent Technologies, Santa Clara, CA, USA) fitted with a binary pump, an online degasser, and an autosampler. The separation of the analytes was achieved with a Poroshell 120 EC-C18 column (3.0 × 100 mm^2^, 2.7 µm) (Agilent Technologies, Santa Clara, CA, USA).

The mobile phase composition was water (A) with 0.1% formic acid and acetonitrile (B). The gradient elution method was as follows:

- 0–0.5 min, 5% B;
- 0.5–2 min, 25% B;
- 2–4 min, 50% B;
- 4–6 min, 75% B;
- 6–10 min, 95% B;
- 10–16 min, 5% B.

The last part was performed to restore equilibrium to the column. The temperature of the column was maintained at 35 °C, the flow rate at 0.4 mL/min, and the injection volume at 10 µL.

#### 2.2.2. Q-TOF-MS Conditions

Mass spectrometry was carried out using the Agilent 6550 iFunnel Accurate-Mass Q-TOF-MS instrument and Agilent Dual Jet Stream Electrospray Ionisation (Dual AJS ESI) ion source (Agilent Technologies, Santa Clara, CA, USA).

Mass spectrometry analysis was performed using the same source conditions for both the positive and negative Ionization modes. Source parameters used were as follows:

- Drying gas flow: 14.0 L/min;
- Nebulizer pressure: 35 psi;
- Drying gas temperature: 290 °C;
- Sheath gas temperature: 400 °C;
- Sheath gas flow: 12 L/min nitrogen;
- Scan range: *m*/*z* 50–1800;
- Acquisition rate: 1.5 spectra/s;
- Collision energy: 20 eV.

According to the instrument tuning data, mass errors for all reference ions were within ±0.25 ppm. Data acquisition was controlled using Agilent MassHunter Acquisition Software (Version A.09.00), and the data were processed using MassHunter Qualitative Analysis Software (Version B.07.00) (Agilent Technologies, Santa Clara, CA, USA).

### 2.3. Compound Annotation and LC-Q-TOF-MS Data Processing

High-resolution accurate mass (HRAM) data analysis was conducted via compound annotation based on the Agilent METLIN Metabolomics Database, relevant spectral library, and METLIN Accurate-Mass Personal Compound Database and Library (METLIN_AM_PCDL). Results that were obtained in both positive and negative modes were merged at a per-sample basis. If a compound is observed from a particular commercial product but has been recorded from different analyses or ionization modes, the compound is counted only once to produce a unique compound annotation list for each sample. Due to this process of data analysis, a total of 1414 unique annotation entries were generated for the six different products under study. This figure reflects the extent of the automatic LC-Q-TOF-MS annotation output, and it cannot be assumed that this figure implies that 1414 unique compounds pertaining to olive leaves or biologically validated have been discovered. A presence/absence matrix was constructed by representing the presence of a certain compound in the annotation list of a sample with 1 and its absence with 0. Due to the fact that the annotation of a certain compound is determined through comparison of its spectrum to those of accurate-mass database/spectral library, compounds that have not been validated using an authentic reference standard can be described as putative annotations.

### 2.4. Operational Classification of Phenolic and Phenolic-Related Compounds

However, the definition of “phenolic-related compounds” in this study is not that of a chemical compound class but rather an operational classification for analysis purpose only. Considering the database-derived super-class, class, sub-class, and direct parent information from the compound list derived from LC-Q-TOF-MS, flavonoids, phenolic acids and their derivatives, phenolic alcohols/catechol derivatives, secoiridoids, coumarins, lignans/neolignans, stilbenes, and other phenolics were defined as phenolic or phenolic-related umbrella compounds. By such definition, 288 compounds were classified as phenolic and phenolic-related compounds. It should be mentioned that operational classification does not include structural confirmation of annotations. The number of compounds in each sample was counted for each phenolic compound type, and the average number of compound richness for each phenolic group was calculated for liquid products (S1–S3) and powder/capsule samples (S4–S6). These numbers indicate only the qualitative diversity of compounds and were not considered as peak intensity/peak area/concentration measured by LC-Q-TOF-MS.

### 2.5. Descriptive Evaluation of Product-Group Compound Distribution

The distributions of phenolic and phenolic-related compounds in the liquid and powder/capsule product groups were descriptively compared using a qualitative presence/absence matrix. The number of occurrences of a compound in S1–S3 was calculated as liquid occurrence (0–3), while the number of occurrences in S4–S6 was calculated as powder occurrence (0–3).

The descriptive difference between the two groups was calculated as

Δ = powder occurrence − liquid occurrence

To summarize the distribution within the sample set,

|Δ| = 3 was defined as “very strong”;
|Δ| = 2 as “strong”;
|Δ| = 1 as “weak”.

This classification does not indicate statistical significance and is not intended for the selection of validated biomarkers. A compound was classified as “liquid-only” if it was present in at least one liquid product but not in any powder/capsule products; as “powder-only” if it was present in at least one powder/capsule product but not in any liquid products; and as “shared” if it was present in both product groups. These terms refer solely to the descriptive distribution patterns observed in the six commercial products under investigation.

### 2.6. Targeted Determination of Oleuropein by HPLC-DAD

The compound, oleuropein, was chosen as the target molecule for quantification because it is one of the major marker molecules employed for the standardization and identification of commercial products of olive leaf extract. As such, quantification was conducted separately by HPLC-DAD. The HPLC analysis of oleuropein was performed on an Agilent 1260 Infinity II system with a diode-array detector (DAD). Separation of oleuropein was achieved through a Spherisorb ODS-2 C18 reverse-phase column (4.6 mm × 250 mm, 5 µm, 100 Å; Waters Corporation, Milford, MA, USA) at ambient temperature.

The detection of oleuropein was realized at 233 nm wavelength.

Mobile phase comprised of three parts:

A: water containing 0.2% H_3_PO_4_ (*v*/*v*);
B: methanol;
C: acetonitrile.

The elution was performed in gradient mode at a constant flow rate of 1.00 mL/min. The initial mobile phase composition was 96% A, 2% B, and 2% C. The analytical procedure was adapted from International Olive Council and European Pharmacopoeia procedures [16,17].

#### 2.6.1. Sample Preparation for HPLC

First, 1.0 g of commercial olive leaf extract in solid form and 1.0 mL of commercial olive leaf extract in liquid form were weighed out. They were both prepared using the methanol–water (80:20, *v*/*v*) solvent system, the volume of which was brought to 10 mL. Prepared samples were mixed for 1 h and filtered using a 0.45 µm PTFE membrane filter, followed by injection into the HPLC system. Each commercial product was independently prepared and extracted in triplicate, resulting in three independent extracts for each commercial product analyzed by HPLC-DAD. Conditions of sample preparation were adapted to the commercial formulations, based on the IOC method for phenolics [16] and other published studies [8,18,19,20] on the extraction of phenolic compounds from olive leaves. The hydroalcoholic extraction system methanol–water (80:20, *v*/*v*) was chosen as a good solvent for phenolic compounds and oleuropein; 1 h mixing time was employed to provide standardized solvent–sample interaction for all commercial products under investigation. No additional optimization studies for the solvent system and extraction time were performed.

#### 2.6.2. Calibration and Quantification of Oleuropein

Oleuropein quantitation was performed using the external standard calibration method. Calibration curve was constructed in the concentration range from 10 to 2000 µg/g. Equations of the calibration curve and coefficient of determination (R^2^) are shown in Figure S1. The oleuropein content of samples was calculated by the calibration curve, and the results are shown in µg/g of commercial product and mg/g of commercial product. The calibration curves and HPLC chromatograms of the samples are shown in the Supplementary Materials.

### 2.7. Data Presentation and Descriptive Analysis

As the objective of the study was to identify the chemical fingerprints of six different commercial products, with there being only three different commercial products in each form group (*n* = 3), the comparison of product groups was mainly carried out on a descriptive level. As the samples were not matched pairs and the number of samples within each group did not have enough power to be statistically tested, inferential statistical tests were not performed between the liquid and powder/capsule forms of the products. The LC-Q-TOF-MS data were analyzed based on the qualitative presence/absence of compounds, number of unique compounds, richness of phenolic classes, and occurrence of form-specific compounds. The HPLC oleuropein data were summarized based on the mean ± SD, min and max values of each form, and individual product value. Therefore, the variations among the groups were understood as descriptive trends unique to the particular group of commercial products that were being analyzed rather than statistically proven form effects.

## 3. Results

### 3.1. Overall Chemical Profiling by LC-Q-TOF-MS

The positive and negative ionization information acquired using LC-Q-TOF-MS analysis of six olive leaf commercial samples was merged to develop unique compound profiles of each individual extract. In the case where the same compound appeared more than once within a given extract either in different analytical records or different ionization modes, the compound was taken into account only once. As a result, 294, 419, and 372 annotated compounds were annotated in the liquid extracts (Samples 1, 2, and 3, respectively), whereas 446, 416, and 435 annotated compounds were annotated in the powdered extracts (Samples 4, 5, and 6, respectively).

**Figure 1:**
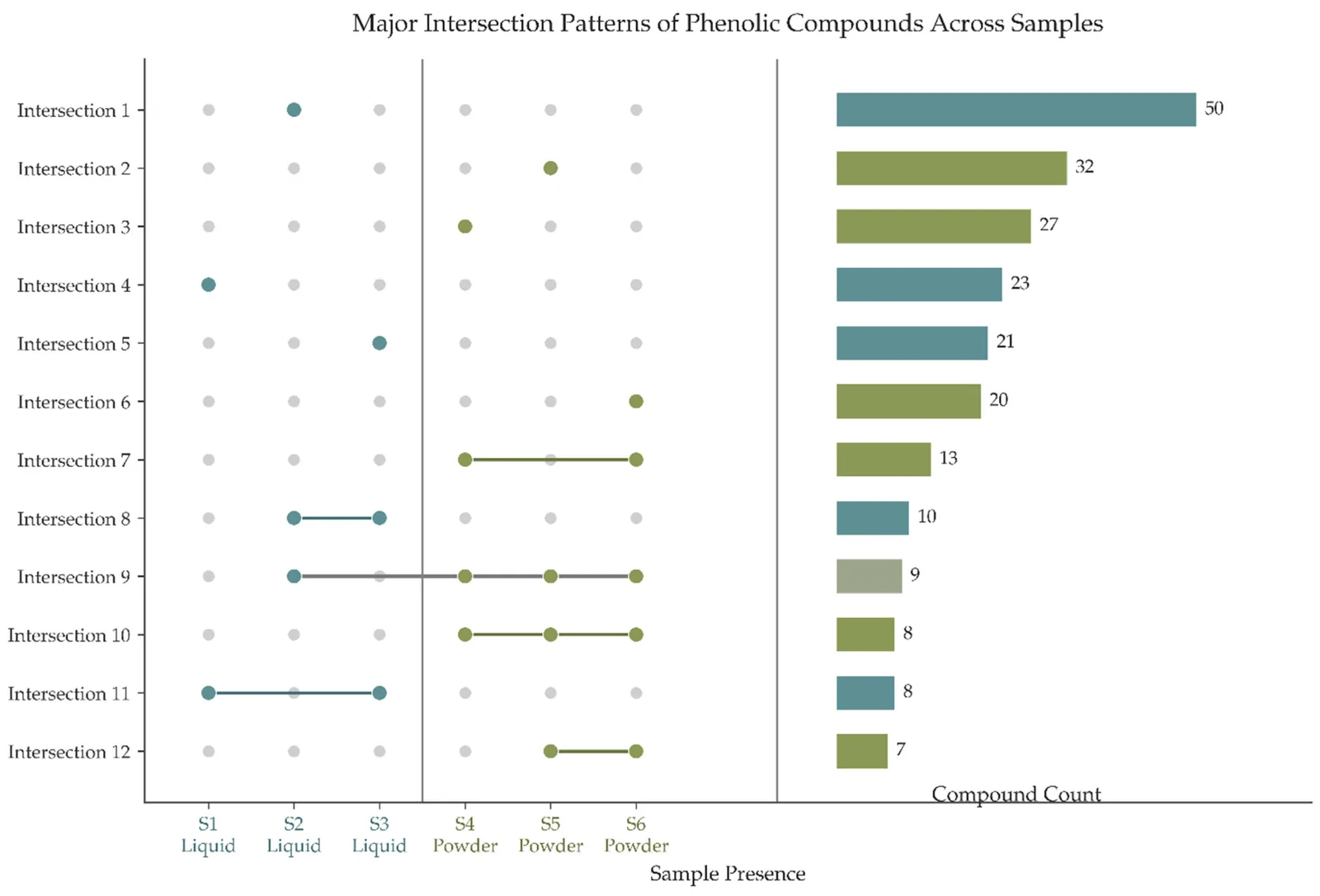
Patterns of major intersections of phenolic and phenolic-related compounds in six commercial olive leaf extracts.

Moreover, 288 putative compounds, which were classified as either phenolic or phenolic-related compounds on the basis of chemical information, were further investigated in more detail. Among these, 114 (39.6 percent) were annotated in at least one sample of the liquid product category only, 113 (39.2 percent) were annotated in at least one sample of the powder product category only, whereas 61 compounds (21.2 percent) were present in at least one sample of both product categories. The complete sample-wise distribution of these 288 putatively annotated phenolic and phenolic-related compounds is provided in Supplementary Table S1. However, the distribution of compounds among samples was not consistent, with many overlap patterns appearing between various sample pairs. The distribution of the biggest overlap categories among S1–S6 samples is presented in Figure 1.

This analysis suggests that commercially available extracts of olive leaves, which are claimed to be extracted from the same source plant, are highly heterogeneous with regard to their overall chemical profiles.

Occupied spaces denote the presence of compounds in each of the extracts, while the lines denote compounds present in more than one extract. Horizontal bars show the number of compounds found in each of the major intersections. S1–S3 are liquid extracts, and S4–S6 are powdered extracts. The liquid and powdered formulations are represented in teal and olive shades, respectively, while the intersections between the two groups of formulations are represented in neutral shades. Compound presence is based on qualitative LC-Q-TOF-MS annotation data.

### 3.2. Descriptive Comparison of Phenolic-Class Richness Across the Investigated Commercial Products

**Figure 2:**
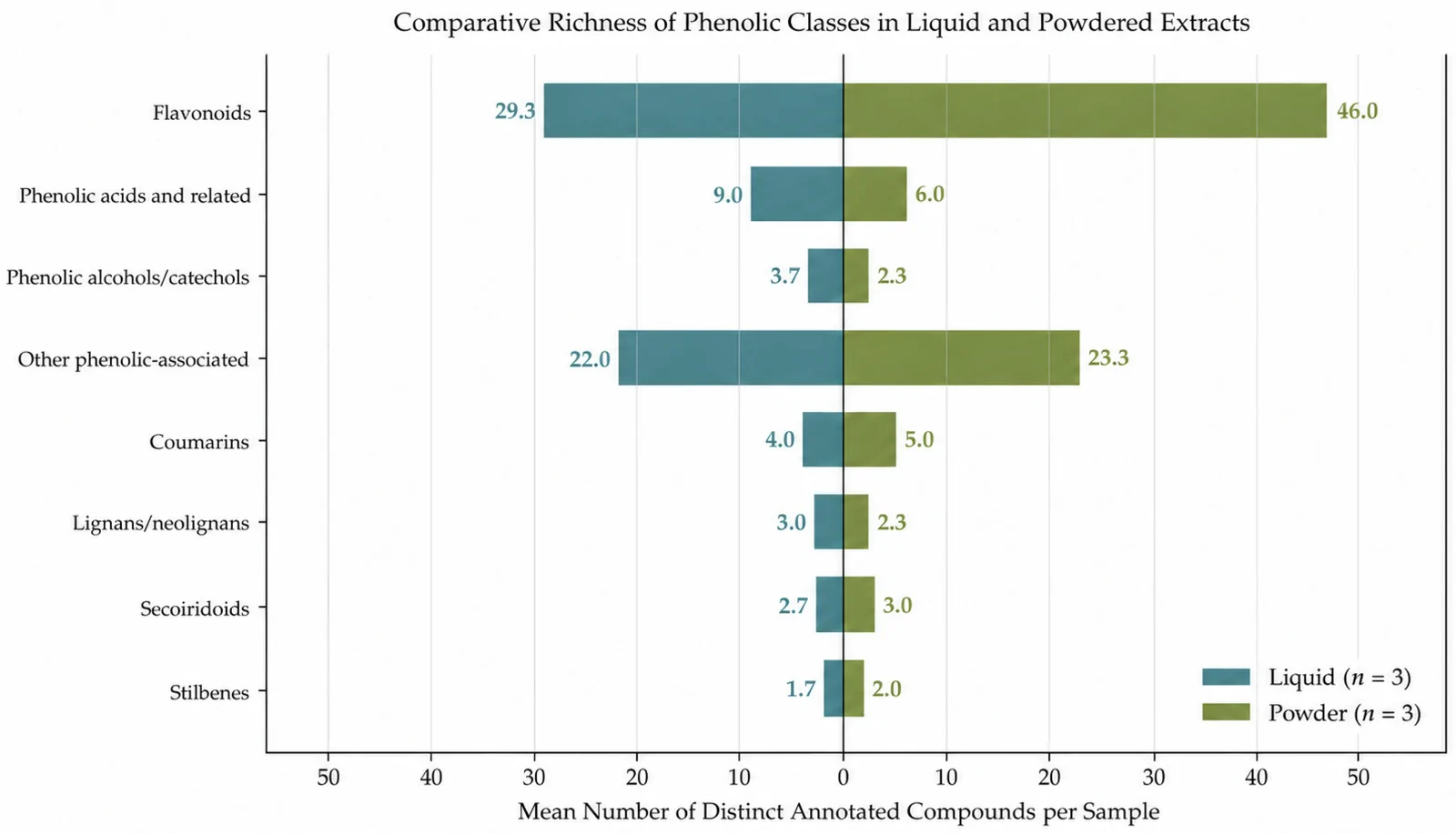
Phenolic and phenolic-related compound richness in liquid and powdered commercial olive leaf extracts.

As for the examination of phenolic and phenolic-related compounds based on their chemical classes, major variation between the liquids and powder commercial samples occurred concerning flavonoids. On average, there were 29.3 flavonoids in each liquid sample; however, the average number in powdered samples equaled 46.0. The average number of phenolic acid analogs was determined to be 9.0 for liquid samples and 6.0 for powder/capsules samples, while phenolic alcohol and catechol derivatives varied to be 3.67 and 2.33, respectively. The average number of other phenolic analogs stayed the same for both categories. The secoiridoid annotation variation was also found to be at the same level for both liquid and powder/capsule products, being 2.67 and 3.00, respectively. The coumarin, lignan/neolignan, and stilbene groups had smaller variations compared to the differences shown by flavonoids (Figure 2). Based on these results, it can be said that in the set of samples considered, commercial powder/capsule products had higher flavonoid annotation richness in LC-Q-TOF-MS. It should be noted that these numbers show only the number of different compounds annotated, not their abundance. The sample-wise chemical profile characteristics and oleuropein contents are summarized in Table 1.

The values indicate the average number of unique compounds annotated in each formulation category (*n* = 3). Liquid extracts are depicted in teal on the left-hand side, while powdered extracts are displayed in olive green on the right-hand side. The numbers have been calculated based on qualitative LC-Q-TOF-MS presence/absence data and refer to chemical richness rather than abundance.

The values for different annotated phenolic compounds, flavonoids, and secoiridoids reflect qualitative compound richness using LC-Q-TOF-MS presence/absence and do not indicate concentration or abundance. The oleuropein concentration was separately analyzed using targeted HPLC.

### 3.3. Targeted Quantification of Oleuropein by HPLC

**Figure 3:**
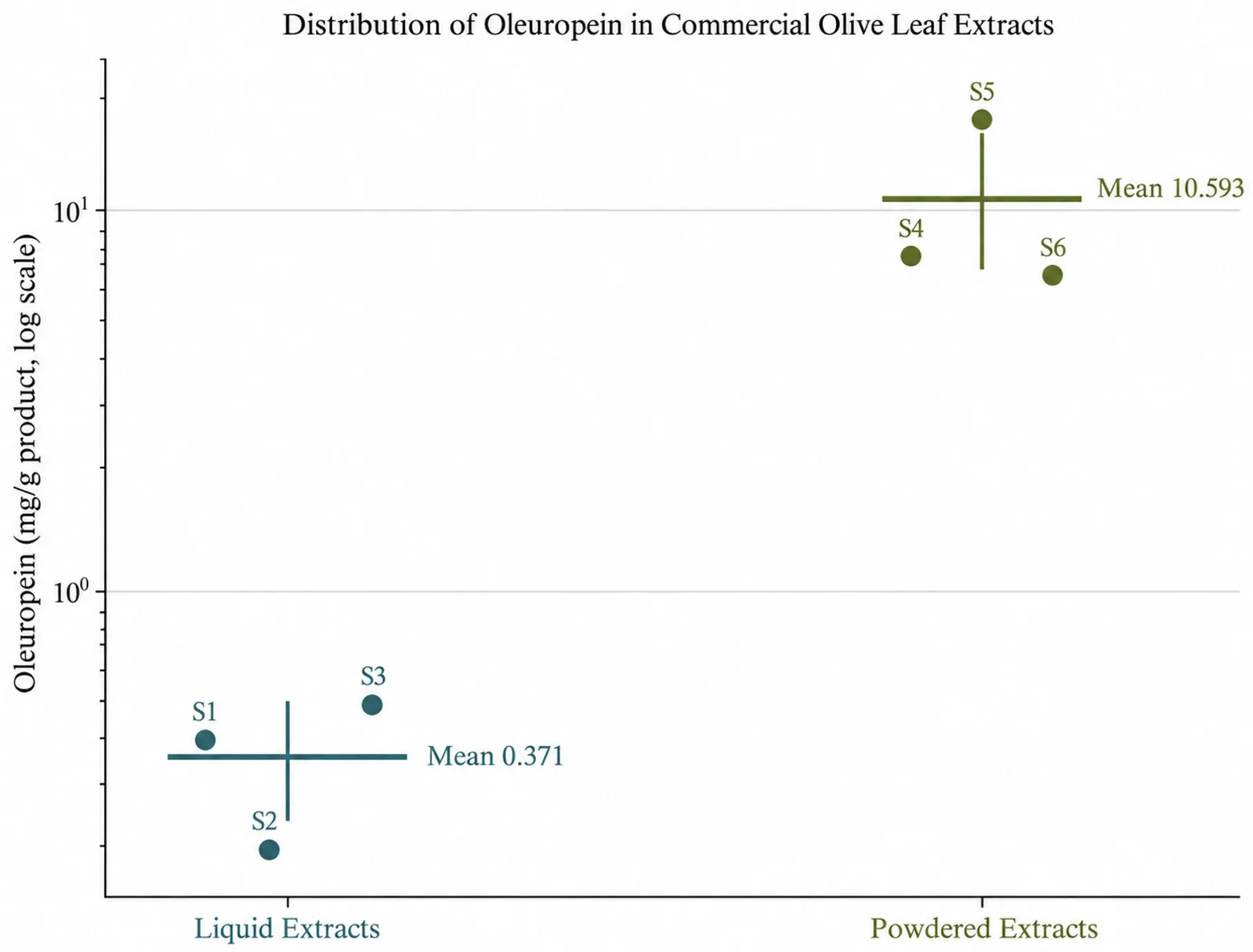
Distribution of oleuropein concentrations determined using HPLC in commercial liquid and powder extracts of olive leaves.

Targeted HPLC studies revealed the presence of oleuropein in all six olive leaf products. Nevertheless, substantial variability of concentrations was recorded among individual products. Oleuropein concentrations in these products varied from 242.0 to 16,283.4 µg/g. Oleuropein content equal to 384.4, 242.0, and 486.5 µg/g was obtained for Sample 1, Sample 2, and Sample 3, while content equal to 8082.7, 16,283.4, and 7412.0 µg/g was obtained for Sample 4, Sample 5, and Sample 6, correspondingly. Mean values of oleuropein in liquid and powder/capsule products were found to be 371.0 ± 122.8 and 10,592.7 ± 4939.7 µg/g, correspondingly. The difference between the maximum and minimum concentration in individual products amounted to about 67-fold (Figure 3). At the same time, the difference between group mean values does not reflect the effect of physical form on the compound content because samples are independent unpaired commercial products. It should be noted that these data are indicative of substantial variability among commercial products.

The individual dots indicate the values for the six different commercially available samples (S1–S6). Horizontal lines represent the mean value of each group, and vertical lines represent the minima–maxima values of each group of formulations. S1–S3 are liquid extracts (teal), whereas S4–S6 are powder extracts (olive green). Concentrations are given in mg/g of the commercially available extract. The *y*-axis scale is presented on a logarithmic basis.

### 3.4. Distribution of Selected Literature-Supported Compounds

**Figure 4:**
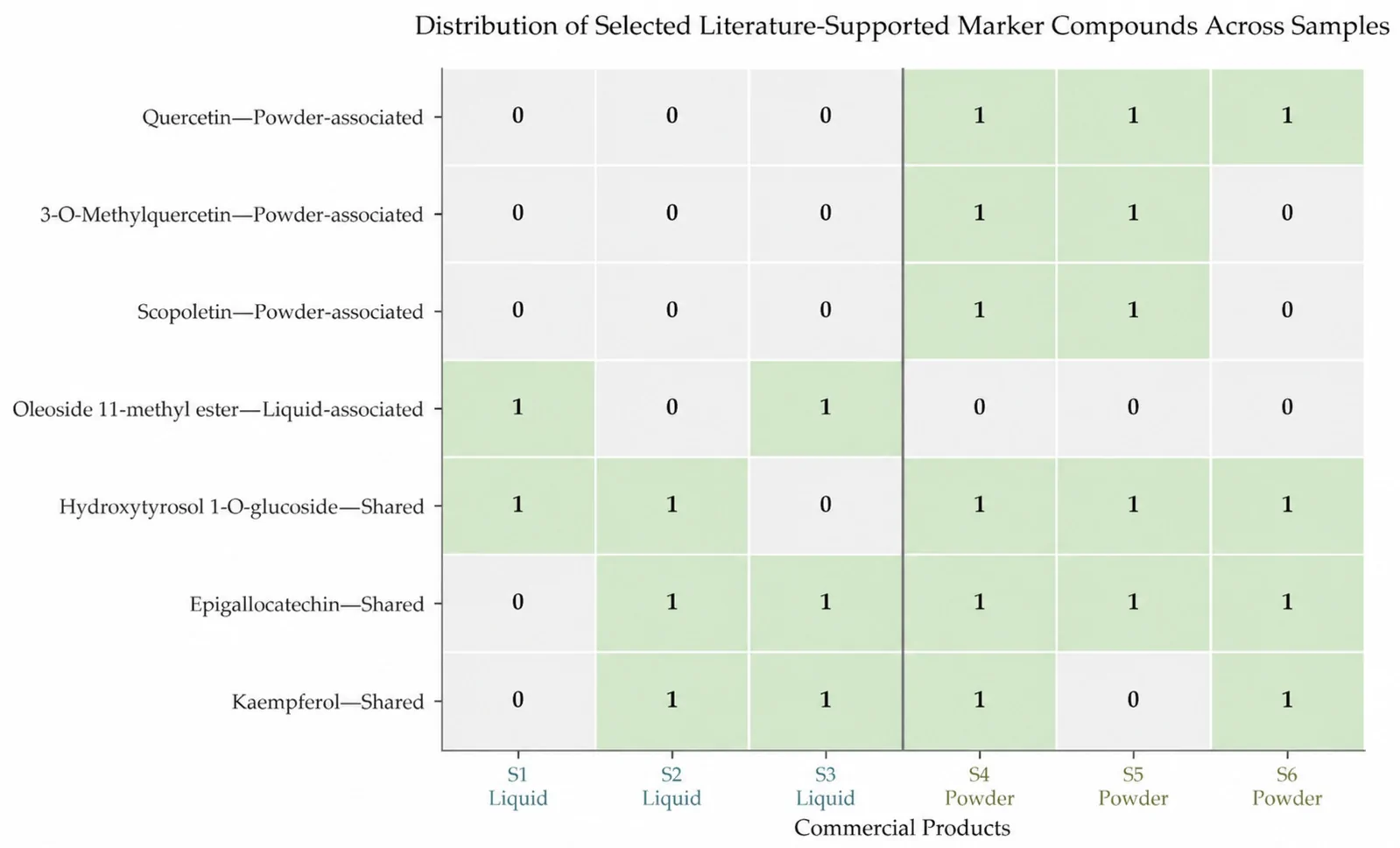
Distribution of selected literature-supported phenolic and secoiridoid compounds in commercial olive leaf extracts.

Since all the compounds annotated in the LC-Q-TOF-MS results may not have equal biological or chemotaxonomic importance, the distribution of some specific phenolic and secoiridoid compounds whose existence is confirmed in the literature on olive leaf was analyzed separately from the rest of the samples. The compound quercetin was observed in all the three powder/capsule products but did not find a place in the annotation list of any liquid product. Similarly, the compounds 3-O-methylquercetin and scopoletin were observed in two powder/capsule products, but none of the liquid products had the annotations for them. Oleoside 11-methyl ester was annotated in two out of the three liquid products, but none of the powder/capsule products had the annotations for this compound. Hydroxytyrosol 1-O-glucoside and epigallocatechin were both annotated in two liquid and three powder/capsule products, and kaempferol was observed in two liquid and two powder/capsule products. These findings demonstrate that the chosen analytes have distinctive occurrence profiles in each of the six commercial products investigated. Nevertheless, such a distribution is descriptive and applies to the present set of samples only, without any implications for marker compounds specific to particular formulations or effects of physical form (Figure 4; Supplementary Table S3). The occurrence frequencies of the selected literature-supported compounds in the two product categories are summarized in Table 2.

The cells show qualitative annotation of compounds using LC-Q-TOF-MS, where “1” indicates annotated compound presence, while “0” indicates compound absence from the annotation list. S1 to S3 are the liquid extracts, while S4 to S6 are powdered extracts. The association tags represent the distribution of the annotated products but not the markers specific to the formulations. The absence of a compound in the annotation list does not mean the absence of the compound itself.

### 3.5. Complementarity of LC-Q-TOF-MS Profiling and Targeted HPLC Analysis

Oleuropein was not included in the final list of compounds generated by the automated LC-Q-TOF-MS annotation workflow. This does not indicate that oleuropein was analytically absent from the samples or that it is absent from mass spectrometric databases; rather, it reflects the output of the specific automated annotation process used in the present study. Targeted HPLC-DAD analysis confirmed the presence of oleuropein in all six commercial products, with varying concentrations among the products. This finding indicates that qualitative profiling by LC-Q-TOF-MS and targeted quantification using authentic standards and calibration are complementary analytical approaches rather than techniques that replace one another. While LC-Q-TOF-MS enabled the assessment of chemical diversity and fingerprint differences among the commercial products, HPLC-DAD allowed for quantitative determination of oleuropein. Together, these two analytical approaches revealed different aspects of the phytochemical heterogeneity among the commercial samples.

## 4. Discussion

### 4.1. Phytochemical Heterogeneity in Commercial Olive Leaf Extracts

The six commercial olive leaf extracts assessed in this study showed distinct LC-Q-TOF-MS-based chemical profiles. The observation that only a limited proportion of the 288 candidate compounds operationally classified as phenolic or phenolic-related compounds occurred in both product groups suggests considerable phytochemical heterogeneity among products marketed under the same botanical source. This finding is consistent with previous studies reporting substantial inter-product variability among commercial olive leaf preparations. Medina et al. [11], Breakspear and Guillaume [21], Garcia-Aloy et al. [22], and Güzelmeriç et al. [23] reported marked differences among commercial products in oleuropein content, phenolic composition, and overall chromatographic profiles. The phenolic composition of olive leaves is influenced by the genotype, harvest time, and environmental conditions [2,3,5,7]. Significant phytochemical differences among cultivars have also been reported [24,25,26], while the cultivar and harvest time may affect the concentrations of oleuropein and other phenolics [27,28], and geographic origin may further contribute to variation in the phenolic composition [29]. Consequently, the differences observed between the liquid and powder/capsule products cannot be attributed solely to physical form. The olive cultivar, raw material composition, extraction procedure, dry matter content, excipients, and other manufacturer-specific practices may all have contributed to the observed chemical profiles.

### 4.2. Differences in Phenolic-Class Richness

The most pronounced difference in phytochemical profiles was observed for flavonoids. The average number of flavonoid annotations per sample was higher in powder/capsule products, whereas the differences were less pronounced for phenolic acids, phenolic alcohols and related compounds, and secoiridoids. However, it should be emphasized that these values reflect chemical diversity rather than compound concentrations. Flavonoids, secoiridoids, simple phenols, and their derivatives constitute major groups of phytochemicals found in olive leaves [2,12,13,30]. LC-MS/HRMS studies have demonstrated considerable diversity in phenolic compounds, as well as differences in phenolic profiles among olive varieties [24,26,30,31]. Nevertheless, the greater diversity of flavonoids observed in powder/capsule products should not be interpreted as evidence that the powder form contains higher concentrations of flavonoids. The extraction of phenolic compounds is influenced by the solvent composition, extraction method, temperature, extraction time, and solid-to-liquid ratio [8,20,29,32,33,34,35,36]. In addition, the stability of phenolic compounds and the recovery of oleuropein can be affected by drying and storage conditions [9,12,37,38]. With this in mind, the observed differences should be interpreted in the context of phytochemical variability arising from the origin of the raw materials and the production and processing conditions of the products (Supplementary Table S4).

### 4.3. Variability in Oleuropein Content Among Products

Targeted HPLC analysis confirmed the presence of oleuropein in all six samples, although substantial differences were observed in its concentration among the samples. The average oleuropein concentration in the commercially available powder and capsule products was 28.6 times higher than that in the liquid products, and more than a 60-fold difference was observed between the samples with the lowest and highest oleuropein concentrations. Wide variations in oleuropein content among commercially available products have also been reported previously [11,21,22,23]. Oleuropein is one of the characteristic secoiridoids of olive leaves [1,2], and its content may be influenced by factors such as raw material characteristics, drying conditions, extraction procedures, and formulation-related factors [9,20,35,36,37,38,39,40]. However, parameters such as moisture content, dry matter content, extract concentration, and excipient composition were not directly comparable among the products. Therefore, these results should not be interpreted as evidence of a causal relationship between product form and oleuropein content; rather, they indicate differences in oleuropein concentrations among the commercially available samples analyzed.

### 4.4. Distributions of Selected Compounds and Complementarity of Analytical Methods

Although quercetin, 3-O-methylquercetin, and scopoletin were annotated exclusively or predominantly in powder/capsule formulations in the current dataset, oleoside 11-methyl ester was annotated only in liquid products. Hydroxytyrosol 1-O-glucoside, epigallocatechin, and kaempferol, however, were annotated in both product categories. The occurrence of these compounds in olive leaves has previously been reported in LC-MS and HRMS studies [13,24,25,26,30,31,41,42,43].

Nevertheless, the observed distribution patterns should not be regarded as definitive markers of the respective product formulations, given the limited sample size and the putative identification of most compounds based on database/library matching. In high-resolution MS analyses, when authentic standards are unavailable, many compounds can only be annotated at a putative level [22,31]. Accordingly, LC-Q-TOF-MS data are more appropriately used for qualitative fingerprinting, and signal intensity should not be interpreted directly as compound concentration. Although oleuropein was not automatically annotated in the current LC-Q-TOF-MS analysis, its detection in all samples by targeted HPLC demonstrated the complementary nature of the two analytical approaches [22,39,44].

### 4.5. Significance and Limitations of the Study

Previous studies have reported variability in oleuropein and other phenolic compounds among commercial olive leaf products [11,21,22,23]. The present study extends these observations by combining LC-Q-TOF-MS fingerprinting with targeted oleuropein analysis in commercial liquid and powder/capsule products, allowing for both marker-specific and broader phytochemical characterization.

However, the study included only six commercial products, with three products in each category, and therefore did not provide sufficient statistical power for inferential group comparisons. The products were also not standardized for factors such as olive variety, harvest conditions, extraction yield, moisture or dry matter content, solvent system, and formulation. In addition, most LC-Q-TOF-MS annotations were not confirmed with authentic reference standards, while targeted quantitative analysis focused on oleuropein because of its established role as a principal marker in the standardization and labeling of commercial olive leaf extract products. Oleuropein concentrations were not normalized to dry matter content. Consequently, the independent effect of product form could not be determined. Future studies should include larger, more standardized sample sets, while targeted quantification of additional phenolic constituents such as hydroxytyrosol, verbascoside, rutin, and luteolin may provide complementary information [35,39].

At the same time, the use of finished commercial products provides a real-world perspective on the chemical variability encountered in the marketplace. Although this variability limits causal comparisons, it complements studies based on standardized laboratory-prepared extracts. From a quality control perspective, the findings also suggest that combining targeted oleuropein analysis with broader chemical fingerprinting may provide more informative product characterization than reliance on a single marker and may contribute to future standardization efforts.

## 5. Conclusions

The present study demonstrated substantial product-to-product variability among commercial olive leaf products in both phytochemical profiles and oleuropein content. LC-Q-TOF-MS fingerprinting revealed differences in the distribution of phenolic compounds, while targeted HPLC confirmed oleuropein in all samples but at markedly different concentrations. Although higher oleuropein levels and greater flavonoid annotation richness were observed in powder/capsule products, these differences cannot be attributed solely to product form because the samples were independent and not standardized for factors such as raw material, extraction conditions, dry matter content, and formulation. Importantly, the study evaluated finished commercial products rather than laboratory-prepared extracts, providing a real-world perspective on the chemical variability consumers may encounter. Overall, combining targeted oleuropein quantification with broader chemical fingerprinting may provide a more informative approach to product characterization and future standardization than assessment based on a single marker alone.

## Figures and Tables

**Table 1:** Chemical profile characteristics determined by LC-Q-TOF-MS and oleuropein content determined by HPLC-DAD in commercially available olive leaf extracts *.

Sample	Form	Distinct Annotated Compounds	Phenolic-Related Compounds	Phenolic-Related Compounds (%)	Flavonoids	Secoi Ridoids	Oleuropein (µg/g)	Oleuropein (mg/g)
S1	Liquid	294	49	16.67	15	3	384.4	0.384
S2	Liquid	419	108	25.78	46	1	242.0	0.242
S3	Liquid	372	69	18.55	27	4	486.5	0.487
S4	Powder	446	92	20.63	46	4	8082.7	8.083
S5	Powder	416	89	21.39	41	3	16,283.4	16.283
S6	Powder	435	89	20.46	51	2	7412.0	7.412

* Oleuropein values represent the mean of three independently prepared extracts for each commercial product.

**Table 2:** Selected literature-based compounds found in the commercial olive leaf extracts examined.

Compound	Chemical Class	Liquid (n/3)	Powder (n/3)
Quercetin	Flavonol	0/3	3/3
3-O-Methylquercetin	Flavonoid	0/3	2/3
Scopoletin	Coumarin	0/3	2/3
Oleoside 11-methyl ester	Secoiridoid	2/3	0/3
Hydroxytyrosol 1-O-glucoside	Phenolic alcohol derivative	2/3	3/3
Epigallocatechin	Flavanol	2/3	3/3
Kaempferol	Flavonol	2/3	2/3

Values represent the number of products that contain each compound in the respective product categories. Distributions pertain only to the six commercial products analyzed and should not be construed as markers for formulations. Compound annotations are tentative unless verified through an authentic standard.

## Data Availability

The original contributions presented in this study are included in the article/Supplementary Materials. Further inquiries can be directed to the corresponding author.

## Supplementary Materials

The following supporting information can be downloaded at: https://www.mdpi.com/article/10.3390/foods15203666/s1, Supplementary Document S1: Calibration Curve and Representative Chromatograms; Supplementary Table S1: Putatively Annotated Phenolic and Phenolic-Related Compounds in Commercial Olive Leaf Extracts; Supplementary Table S2: Presence–Absence Matrix of Distinct Annotated Compounds Across Commercial Olive Leaf Extracts; Supplementary Table S3: Descriptive Screening of Phenolic and Phenolic-Related Compound Annotations; Supplementary Table S4: LC-Q-TOF-MS Annotation and Distribution Dataset for Commercial Olive Leaf Extracts.
