# Supplementary material for: Chemical Characterization and Phenolic Profiling of Commercial Olive Leaf Extracts Using LC-Q-TOF-MS and HPLC-DAD
Source: Foods. 2026 Oct 15;15(20):3666. doi: 10.3390/foods15203666 (PMC13704273; doi:10.3390/foods15203666)
Supplement: Supplementary file 1 [file foods-15-03666-s001.zip › Supplementary Document_S1_Rev.pdf]

Calibration Curve and Representative Chromatograms

1. Calibration and Quantification of Oleuropein

Oleuropein quantitation was performed using the external standard calibration method. Calibration curve was constructed in the concentration range from 10 to 2000 µg/g. Equation of calibration curve and coefficient of determination (R^2) are shown in in Figure S1. Oleuropein content of samples was calculated by the calibration curve, and the results are shown in µg/g of commercial product and mg/g of commercial product. HPLC chromatograms of the samples are shown in Supplementary Materials.

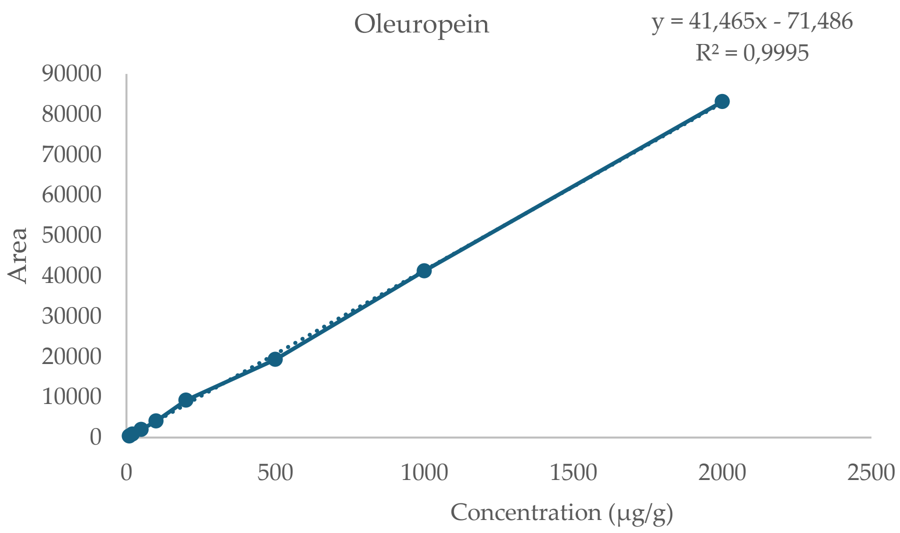

Figure S1. Calibration curve.

2. Representative HPLC-DAD Chromatogram and LC-Q-TOF-MS Total Ion Chromatograms (TICs)

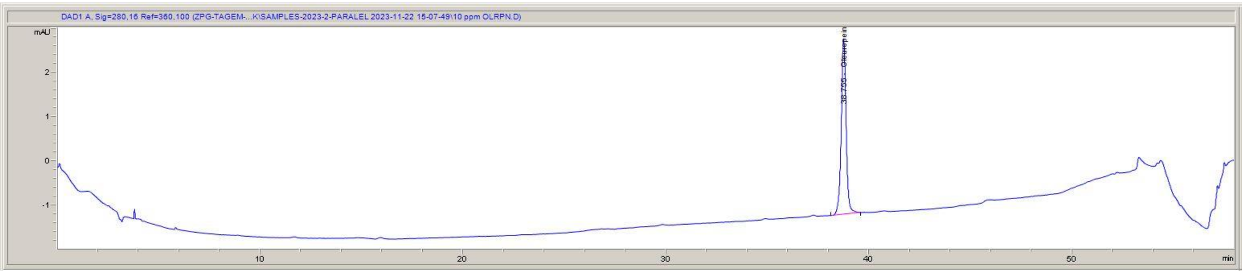

Figure S2. HPLC-DAD chromatogram of the oleuropein reference standard.

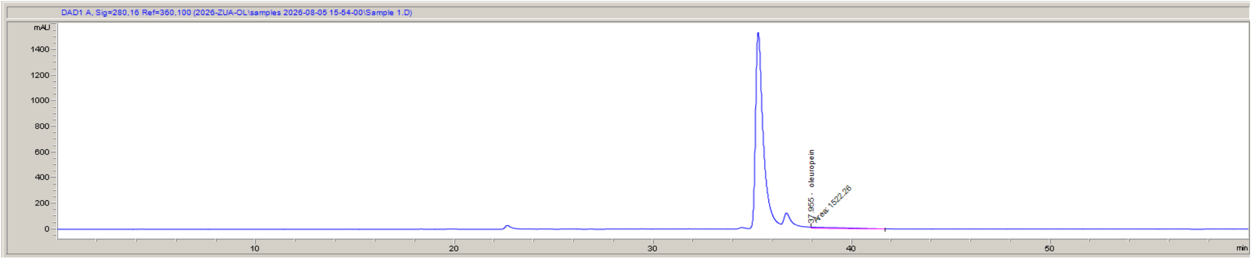

Figure S3. HPLC-DAD chromatogram of Sample 1.

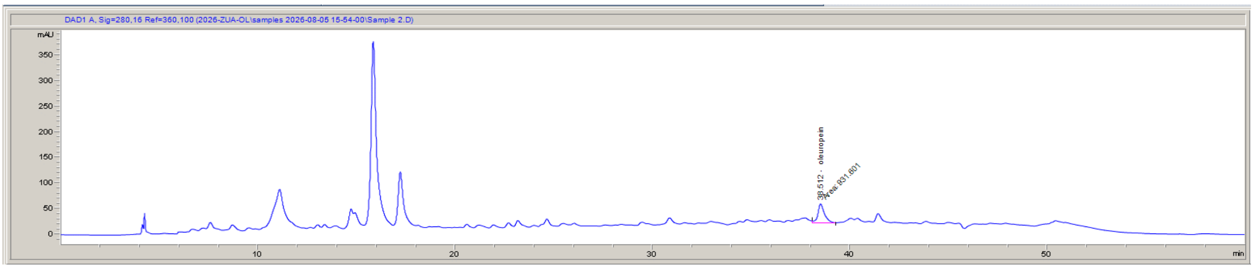

Figure S4. HPLC-DAD chromatogram of Sample 2.

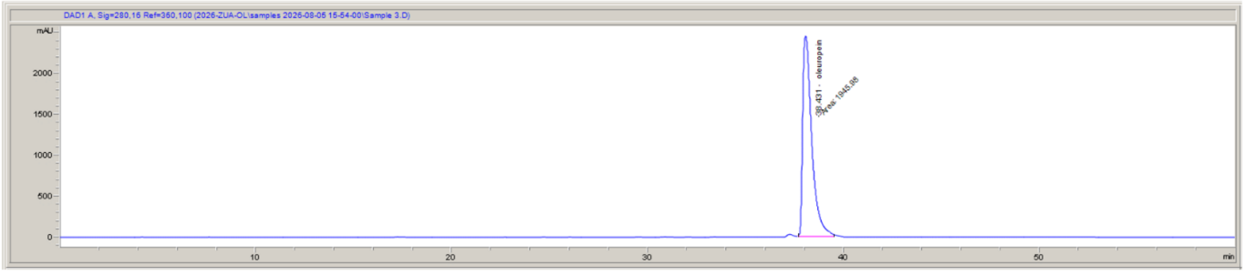

Figure S5. HPLC-DAD chromatogram of Sample 3.

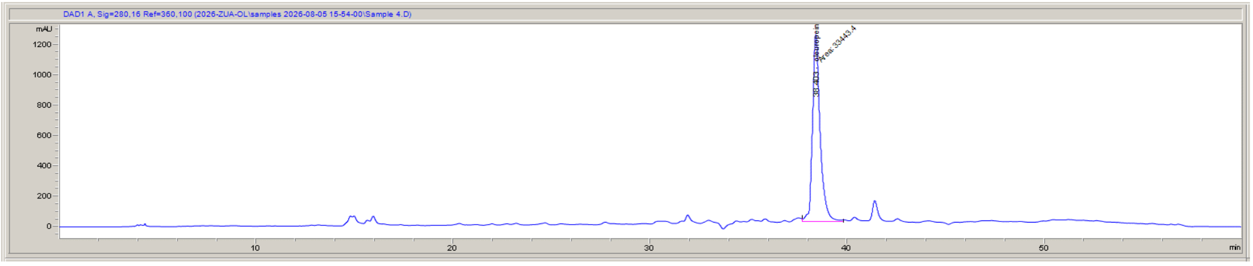

Figure S6. HPLC-DAD chromatogram of Sample 4.

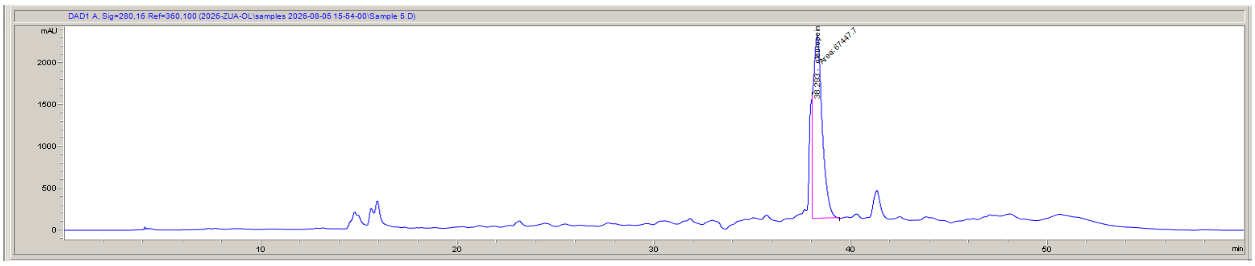

Figure S7. HPLC-DAD chromatogram of Sample 5.

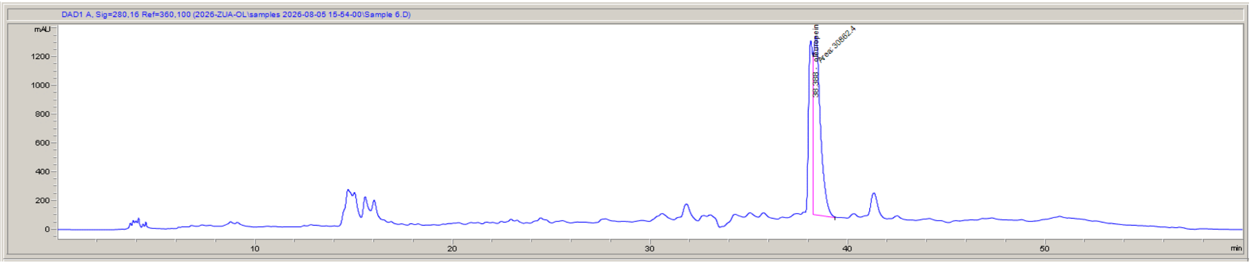

Figure S8. HPLC-DAD chromatogram of Sample 6.

A

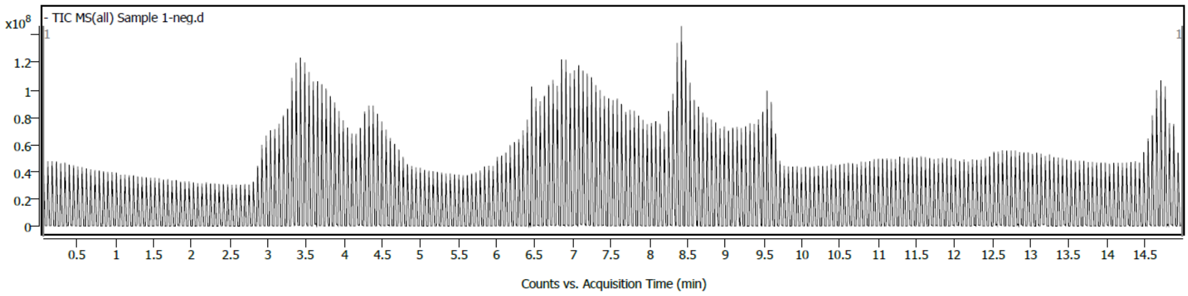

B

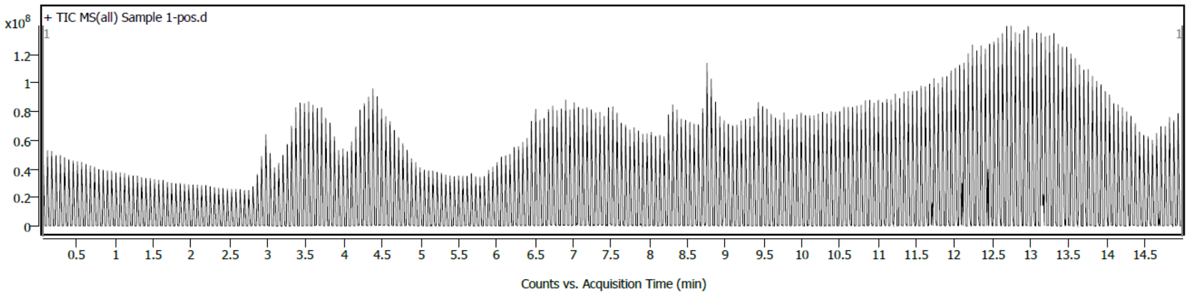

Figure S9. LC-Q-TOF-MS total ion chromatograms (TICs) of Sample 1: (A) negative-ion positive-ion mode and (B) positive-ion mode.

A

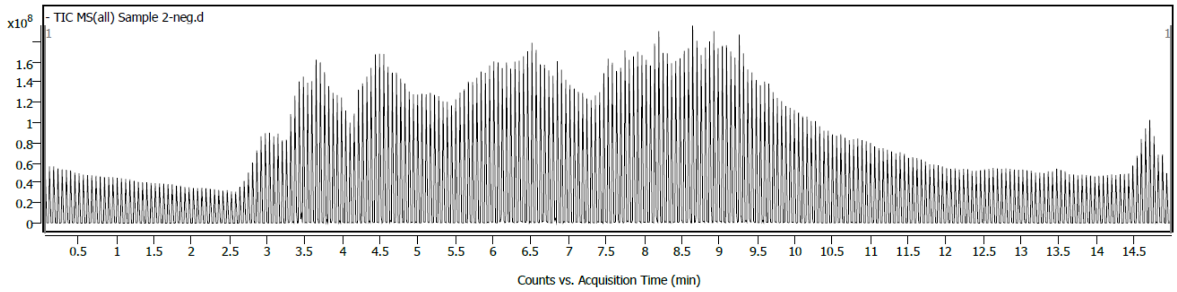

B

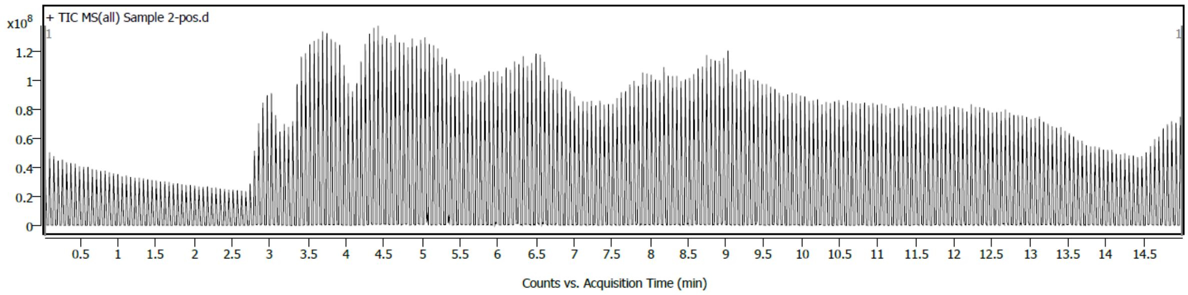

Figure S10. LC-Q-TOF-MS total ion chromatograms (TICs) of Sample 2: (A) negative-ion positive-ion mode and (B) positive-ion mode.

A

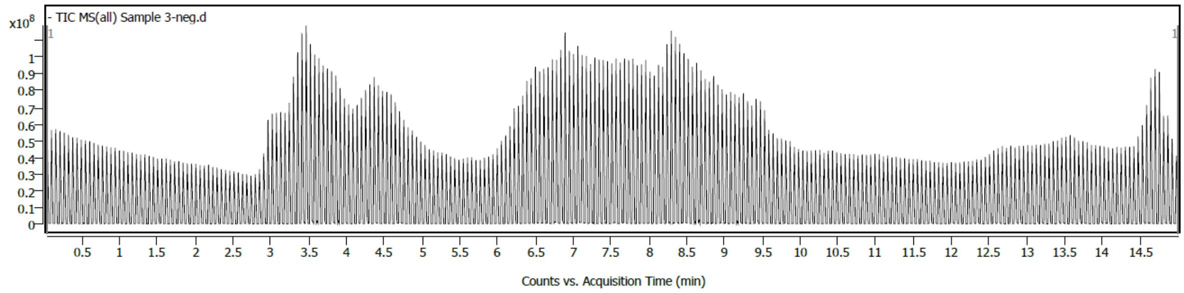

B

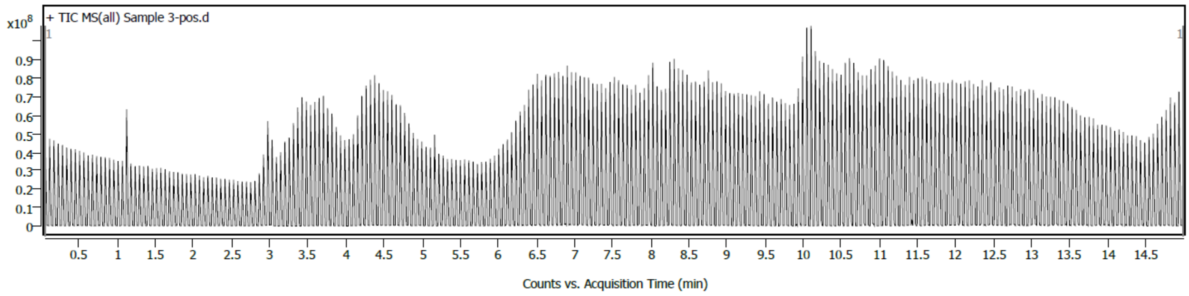

Figure S11. LC-Q-TOF-MS total ion chromatograms (TICs) of Sample 3: (A) negative-ion positive-ion mode and (B) positive-ion mode.

A

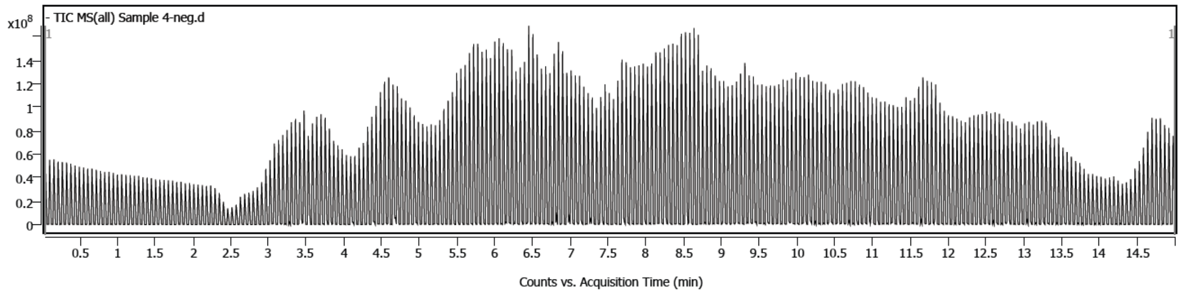

B

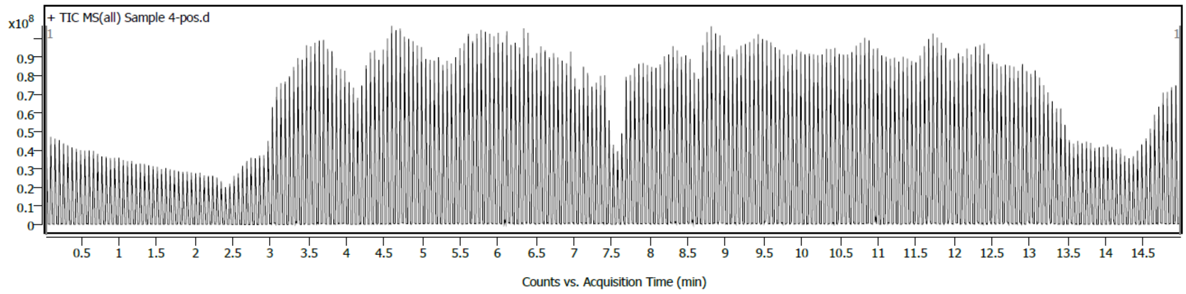

Figure S12. LC-Q-TOF-MS total ion chromatograms (TICs) of Sample 4: (A) negative-ion positive-ion mode and (B) positive-ion mode.

A

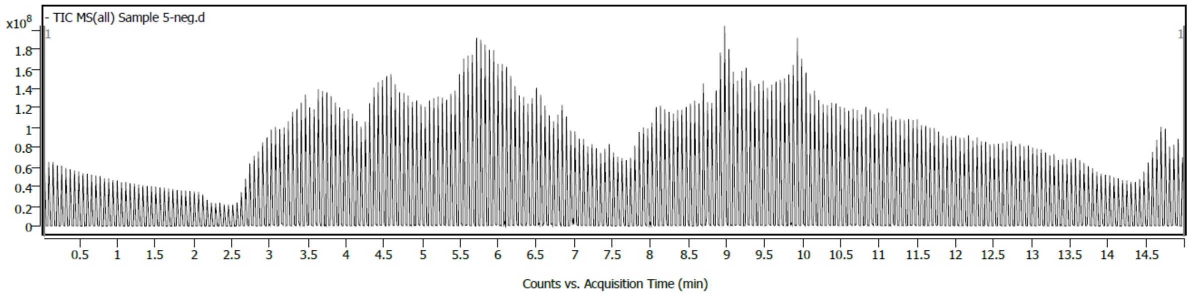

B

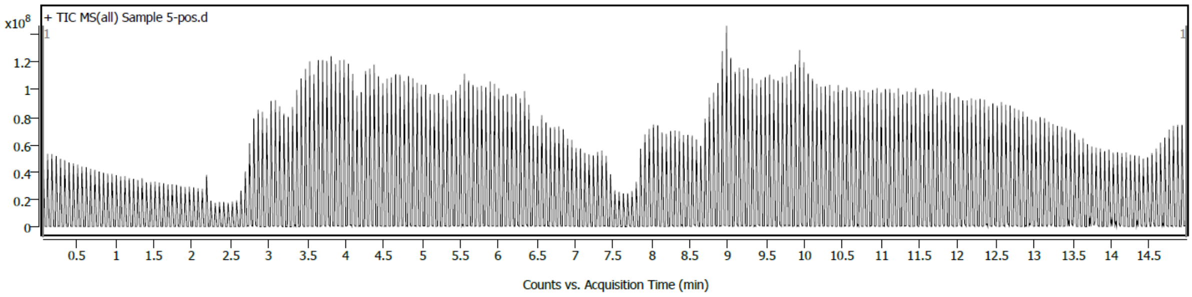

Figure S13. LC-Q-TOF-MS total ion chromatograms (TICs) of Sample 5: (A) negative-ion positive-ion mode and (B) positive-ion mode.

A

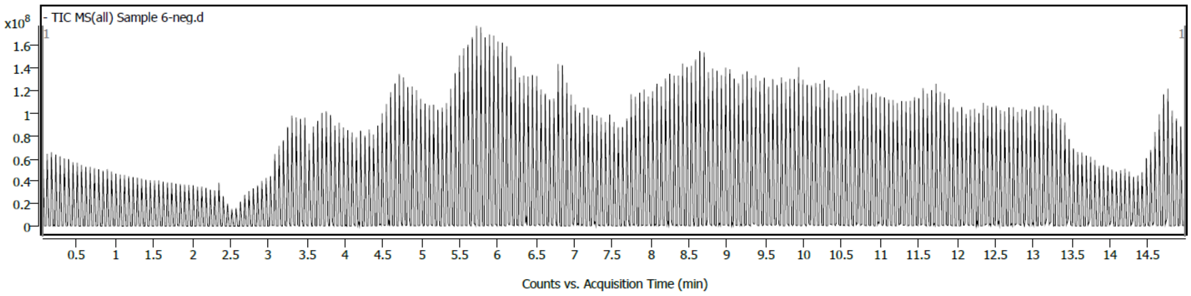

B

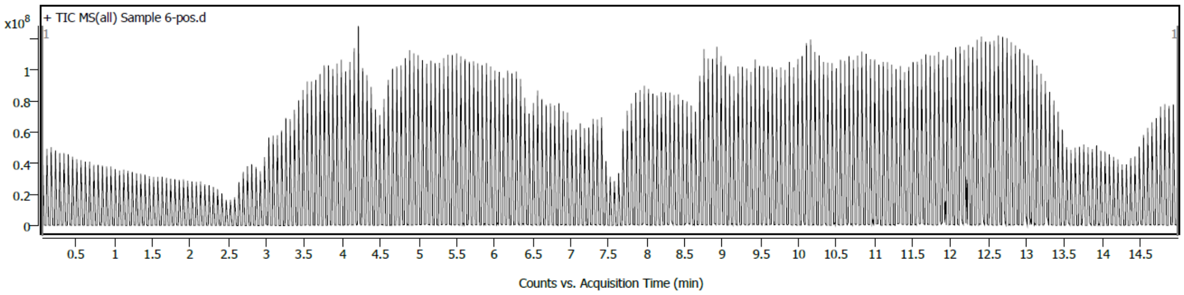

Figure S14. LC-Q-TOF-MS total ion chromatograms (TICs) of Sample 6: (A) negative-ion positive-ion mode and (B) positive-ion mode.
